# Fine-scale spatial mapping of urban malaria prevalence for microstratification in an urban area of Ghana

**DOI:** 10.1186/s12936-025-05724-9

**Published:** 2025-12-18

**Authors:** Samuel Kweku Oppong, David Kwame Dosoo, Nana Yaw Peprah, George Asumah Adu, Wahjib Mohammed, Jennifer Rozier, Kingsley Kayan, Michael McPhail, Punam Amratia, Kefyalew Addis Alene, Kwaku Poku Asante, Peter W. Gething, Keziah L. Malm

**Affiliations:** 1 School of Population Health, Curtin University, Kent Street, Bentley, WA 6012 Australia; 2 Malaria Atlas Project, The Kids Research Institute Australia, Perth Children’s Hospital, 15 Hospital Avenue, Nedlands, WA 6009 Australia; 3 National Malaria Elimination Programme, Ghana Health Service, 2nd Morocco Lane, Off Independence Avenue, Ministerial Area, 233 Accra, Ghana; 4 Research and Development Division, Kintampo Health Research Centre, Ghana Health Service, P.O. Box 200, Kintampo, Ghana; 5 Ifakara Health Institute, Plot 463 Kiko Avenue Mikocheni, Dar es Salaam, Tanzania

**Keywords:** Urban, Malaria, Prevalence, Geospatial, Bayesian, Stratification

## Abstract

**Background:**

Malaria is a focal disease and more localized in low endemic areas. The disease is increasingly becoming a concern in urban areas in most sub-Saharan African countries. The growing threats of *Anopheles stephensi* and insecticide resistance magnify this concern and hamper elimination efforts. It is, therefore, imperative to identify areas, within urban settings, of high-risk of malaria to help better target interventions.

**Methods:**

In this study, a set of environmental, climatic, and urban covariates were combined with observed data from a malaria prevalence study in Ghana and geospatial methods used to predict malaria risk in the Greater Accra Region of Ghana. Georeferenced data from 12,371 surveyed children aged between 6 months and 10 years were included in the analysis. The probability of malaria prevalence exceeding 10% (exceedance probability) in the Region was further calculated.

**Results:**

Predicted malaria prevalence in this age group ranged from 0 to 49%. Satellite-driven data on tasselled cap brightness, enhanced vegetation index and a combination of urban covariates were predictive of malaria prevalence in the study region. A map that quantified the probability of malaria prevalence exceeding 10% was produced.

**Conclusions:**

The malaria prevalence and exceedance probability maps showed areas within the districts earmarked for malaria elimination that have high malaria risk. It is anticipated that this study results can support decision making at both national and subnational levels on deployment of strategic malaria interventions.

**Supplementary Information:**

The online version contains supplementary material available at 10.1186/s12936-025-05724-9.

## Background

Malaria remains a critical global health challenge, with an estimated 247 million cases and over 600,000 deaths reported annually, the vast majority in sub-Saharan Africa [1]. Although significant strides have been made in reducing the burden of malaria over the past two decades, progress has slowed in many regions. Malaria has historically been considered a rural disease, closely linked to natural water sources and vector habitats [2]. However, urban malaria is becoming an increasing concern, particularly in malaria-endemic regions, such as Ghana, where rapid urbanization has introduced new challenges for disease control [3]. Urbanization is a global phenomenon, with over half of the world’s population now residing in urban areas, a figure that is expected to rise to 68% by 2050 [4, 5]. In sub-Saharan Africa, the urban population has surged from 39% in 2003 to 53% in 2021 [6]. This rapid, and often unplanned, urbanization has led to the proliferation of informal settlements and slums resulting in fundamental socioeconomic challenges [2, 7, 8]. In 2020, slum dwellers in Africa accounted for 51.3% of urban populations, and these areas tend to be characterized by overcrowding, poor sanitation, and insufficient access to healthcare services [9, 10]. These factors foster environments conducive to malaria transmission, particularly in the absence of effective vector control strategies [3].

Malaria transmission is highly heterogenous across endemic countries and recent studies have reported the growing fraction of malaria burden emerging from urban areas since 2003 [2]. Urban malaria control faces emerging threats such as increasing insecticide resistance and the invasion of *Anopheles stephensi*, a mosquito species adept at urban breeding. Again, as areas enter low transmission settings, malaria become more focal, and this requires interventions that are deliberately targeted at hotspots of local transmission and prevent importation of cases [11]. Additionally, urban populations tend to have lower immunity due to limited prior malaria exposure, further complicating control efforts [1]. To address the stalling progress in malaria reduction in many countries, while recognizing the transmission dynamics of malaria in urban areas, the World Health Organization (WHO) recently launched a global framework to respond to urban malaria [12].

The Ghana National Malaria Programme (NMP) has transitioned from a control program to an elimination programme in 2022 [13], aiming to accelerate malaria elimination efforts by targeting interventions based on local burden. A key component of this approach was a national malaria burden stratification exercise, which identified 21 districts in the Greater Accra Region, the capital of Ghana as sustaining very low transmission and thus prioritized for malaria elimination [14]. The Region has over 70% of the districts classified as urban and the rest as peri-urban interspersed with rural settlements [15]. The stratification exercise relied heavily on malaria case reporting data from health facilities and district-level model-based malaria prevalence estimates. This approach provided important information at the district level but did not capture the fine-scale spatial heterogeneity of malaria transmission within districts. This intra-district variation in risk is relevant, particularly in urban areas where transmission is often highly focal, driven by a complex mix of socio-economic conditions, varying levels of infrastructure, and diverse landscapes [3].

Understanding these micro-level variations in risk is important for guiding local malaria control efforts and ensuring that interventions are precisely targeted to areas where they are most needed [12]. Fine-scale spatial mapping of malaria prevalence can provide the granular information necessary to identify malaria hotspots within urban areas and better understand the factors contributing to high-risk areas. This study, therefore, aimed to provide a fine-scale malaria risk map for the Greater Accra Region to enhance malaria programming. Specifically, to (1) identify differences in malaria risk within districts (sub regional administrative areas, (2) determine factors associated with malaria prevalence in urban settings), and (3) provide actionable data to guide local planning for targeted intervention strategies within this major urban area of Ghana.

## Methods

### Study area

The study was conducted in the Greater Accra Region which is home to Ghana’s national capital—Accra. It is the smallest of Ghana’s 16 administrative regions by land size but the most populous, with approximately 5,500,000 inhabitants in 2021, representing 18% of the country’s population [16]. Greater Accra Region is highly urbanized with 92% of its population living in urban areas. The Region is divided into 29 administrative local government areas (LGAs), which comprise of districts, municipals, and metropolis, based on population sizes [17]. The high cost of living [18] has also contributed to the development of several slum areas within the region [19].

Geographically, Greater Accra is bordered by the Eastern Region to the north, the Volta Region to the east, the Central Region to the west and the Atlantic Ocean to the south. The Region lies within the dry coastal equatorial climatic zone with temperatures between 20^0^ and 30 ^0^C, an ideal temperature for malaria vector and parasite development [20]. Annual rainfall ranges from 635 mm along the coast to 1140 mm in the northern part with two notable rainfall peaks in June and October [16]. Two main rivers, the Volta and Densu, flow through the Region in addition to smaller streams that flow seasonally from the Akwapim Ridge in the Eastern Region, through lagoons, into the sea.

**Fig. 1:**
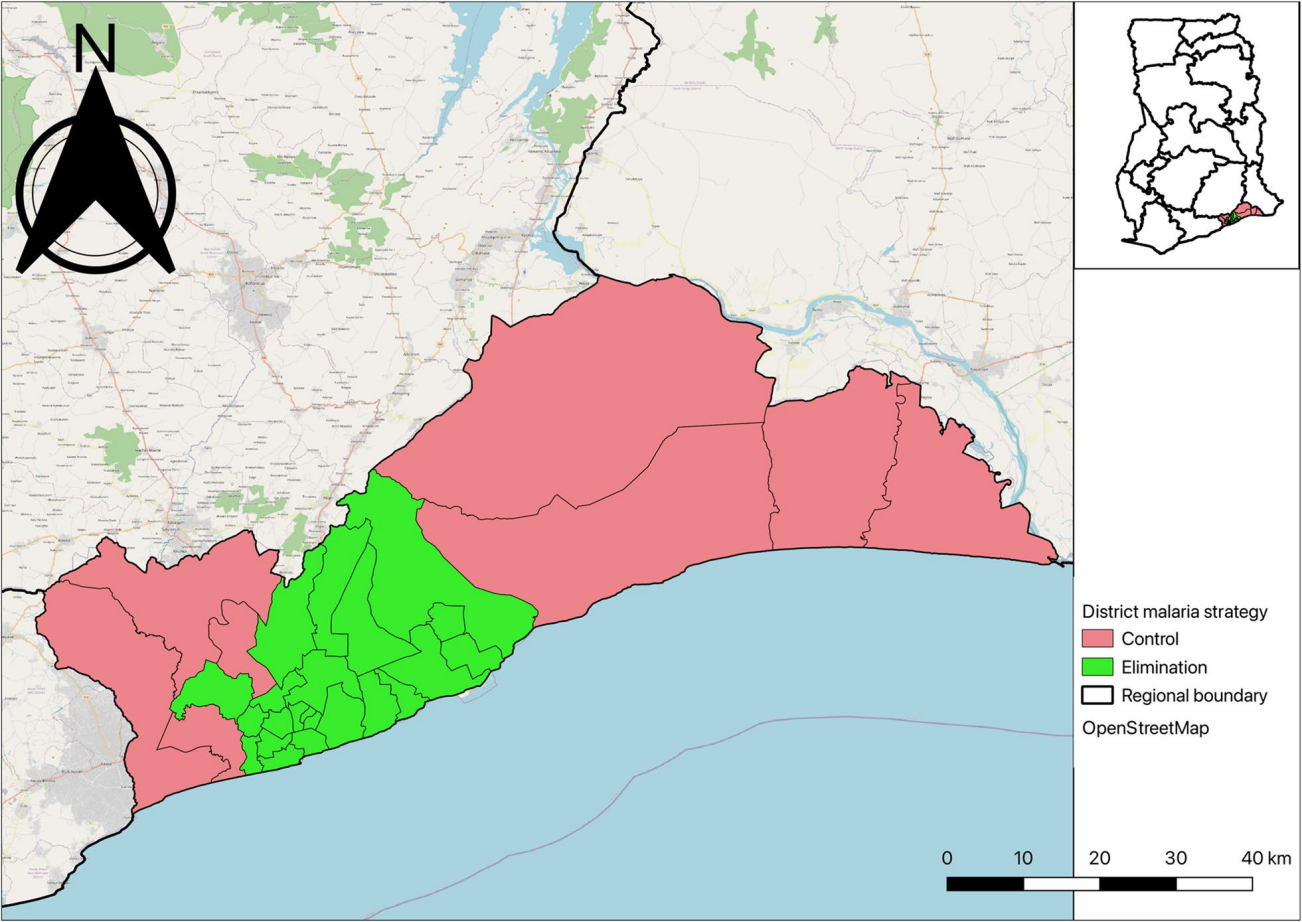
Map of Greater Accra showing districts earmarked for malaria elimination and control

The Region has the lowest malaria risk out of the 16 regions in Ghana with a *Plasmodium falciparum* parasite prevalence rate among children under 5 years of 2% according to the 2022 Ghana Demographic and Health Survey (GDHS) [21]. Malaria is perennial in the region with a bimodal peak in June and October. Figure 1 shows the LGAs in the region and areas earmarked for malaria elimination by the National Malaria Elimination Programme (NMEP).

### Data

Data for this study was sourced from a cross-sectional household survey conducted across all 29 Districts of Greater Accra in 2020 [22] by the Kintampo Health Research Centre, Research and Development Division and the National Malaria Programme of the Ghana Health Service [22]. The study population included children aged 6 months–10 years, with the inclusion criterion of being resident in the community for at least 3 months preceding the survey. This age group (6 months–10 years) is one of the most at risk populations in malaria endemic countries due to several factors including low immunity [23]. Within each district, a maximum of three subdistricts were randomly selected. In each selected subdistrict, all communities were listed and assigned numbers from which 14 communities were selected using systematic random sampling. Within the selected communities, households were selected by systematic random sampling as teams moved along directional lines, determined randomly from a central point. In each household, a maximum of two children aged 6–59 months and up to two children aged 5–10 years, eligible, were enrolled. For further details on the sampling strategy see Dosoo et al*.* [22]. The survey was conducted in October after COVID-19 restrictions had been eased and coincided with the second peak malaria season in the region.

Trained survey teams administered a structured questionnaire to caregivers of eligible children to collect information on demographics, household characteristics and the use of malaria interventions. Additionally, each child underwent a rapid diagnostic test (RDT) specific for *Plasmodium falciparum* after receiving caregiver consent, with test results recorded as either positive or negative for *P. falciparum*. Ten percent (10%) of the sampled children were also sampled for microscopy testing for quality control purposes.

The dataset used for this analysis included RDT test result for each child and demographic attributes of the child such as age, sex, and place of residence. Out of the 17,035 records in the dataset, 1378 had missing geocoordinates, while 3257 were assigned geocoordinates at the centroid of the community they reside. Only 12,371 had coordinates at the household level and were included in the analysis (supplementary Fig. 1). The final dataset had at least one child in each household with approximately 86% of households having only one child.

Permission to use the data set was obtained from the National Malaria Elimination Programme (NMEP) and Kintampo Health Research Centre of Ghana. The original study received ethical approvals from the Scientific Review Committee and Institutional Ethics Committee of the Kintampo Health Research Centre (KHRC).

### Patient and public involvement

Patient and Public were not involved in this research since it was a secondary analysis from the original study.

#### Variables

The dependent variable in this analysis was *P. falciparum* infection derived from the RDT test results, a binary outcome coded as 0 for negative and 1 for positive.

Independent variables were selected based on evidence of association with malaria infection from previous studies and the availability of high-resolution raster files for the study settings [24]. For this analysis, 19 high-resolution remote sensing variables were considered for this analysis and categorized into three main broad classes—landcover, environmental/climatic, and urban (supplementary Table 1). The details of how the covariates were extracted are outlined in supplementary methods section.

#### Data/covariate cleaning

Data cleaning and analysis were conducted using R software, version 4.3.2 [25]. Covariate rasters were cropped and masked to match the shapefile boundaries of the study region. Appropriate transformations were applied to standardize the rasters. The mean values of the rasters were then extracted for all points in the dataset for analysis.

#### Exploratory analysis

An exploratory analysis was conducted to understand the relationships between the malaria prevalence and the suit of explanatory covariates. Firstly, a univariate logistic regression analysis was performed to identify covariates significantly associated with the dependent variable. Additionally, a non-spatial multivariate logistic regression model was used to assess the combined effect of all covariates on the dependent variable. The building height covariate was excluded due to its extremely high odds ratio and confidence intervals in the multivariate model (Supplementary Table 2). Crops and flooded vegetation were also excluded because they showed some spatial patterns in the predicted maps which were not consistent with the data. Point coordinates of the observations were then plotted on the map of the study area to visualize the spatial distribution of the RDT test results (Supplementary Fig. 2).

#### Variable selection

The inclusion of urban covariates at 100 m resolution was to account for the landscape heterogeneity we find in typical urban areas in Africa in the prediction. In order not to lose the full effect of the remaining four urban covariates in the model, their effects were combined through a principal component analysis (PCA). The PCA enabled us to transform the high-dimensional, correlated urban covariates into a low-dimensional space that captures the relevant information needed [26]. The rasterPCA() function in R [27] was used for the PCA analysis, after which a sensitivity analysis was conducted to determine which components improved model fit. Three principal components (PC1, PC2, and PC3) that explained more than 99% of the variance in the data were selected (Supplementary Table 3). PC1-3 therefore, replaced the four urban covariates in the suite of 18 covariates considered during variable selection.

In testing for multicollinearity among the covariates. The variable inflation function (VIF) was used and covariates with a VIF over 23 sequentially dropped until all remaining covariates had a VIF < 23. This led to the removal of NDVI and LST band 10, and TCG with 12 covariates retained. See Supplementary Table 4 for the final set of covariates that were included in the model fitting.

### Geostatistical model

The geostatistical model used in this analysis assumed that the probability $p$ of a child ${x}_{i}$ testing positive for *P. falciparum* malaria at a location $i=1,\dots ,n$ follows a binomial distribution, expressed as:

$${Y}_{i}|p\left({x}_{i}\right) \sim Binomial({N}_{i},p\left({x}_{i}\right))$$

where ${Y}_{i}$ represents the number of children testing positive at a location $i$ out of ${N}_{i}$ children tested. Malaria prevalence was modelled at household level and a binomial distribution chosen because of its flexibility. Even though most households have only one child, others have multiple children, therefore, a proportion positive can be derived. The binomial model with logit link permits to simultaneously model both households with one child and those with more than one.

The binomial geostatistical Model is formulated using the logit transform as:

$$logit\left(P\left({x}_{i}\right)\right)=\mathrm{log}\left(\frac{P\left({x}_{i}\right)}{1-P\left({x}_{i}\right)}\right)= {\beta }_{0}+{\beta }_{n}{X}_{n}+S({x}_{i})$$

where  ${\beta }_{0}$ is the intercept parameter, ${\beta }_{n}$ is the vector of covariate coefficients for the covariates in the design matrix ${X}_{n}$, and ${S(x}_{i})$ is the spatial random field, which represents the spatial variation between the sampled locations. The spatial random field was assumed to have a zero-mean Gaussian process with Matérn covariance function. The covariance between the two points located at ${x}_{i} and {x}_{j}$ is given by;

$$COV(d)=\frac{{\sigma }^{2}}{{2}^{\nu -1}\Gamma \left(\nu \right)}(k\parallel d\parallel {)}^{\nu }{K}_{\nu }(k\parallel d\parallel )$$

where $d$ is the distance between location ${x}_{i} and {x}_{j}$; ${\sigma }^{2}$ is the variance; ν is the smoothness parameter that determines the analytic smoothness of the process;${K}_{\nu }\left(\cdot \right)$ is the modified Bessel function of second kind and order $\nu >0$;$k>0$ is the range parameter which determines how quickly the covariance decays as distance increases; and $\Gamma (\nu )$ is the Gamma function.

### Model fitting

The model for this analysis was fit using the Integrated Nested Laplace Approximations (INLA) package in R, version 24.02.09. The R-INLA package has been shown to be a computationally efficient alternative [28, 29] to the Markov chain Monte Carlo (MCMC) method, particularly when combined with the Stochastic Partial Differential Equation (SPDE) approach [30]. The combination of INLA and SPDE facilitates efficient point-level data analysis within a Bayesian framework [31, 32]. To begin, a mesh surface was constructed across the boundary of the study region and an SPDE model built to generate the index set. Also, a projection matrix was built that will project the mesh, on which the spatial random field is represented, to the observed locations. Finally, the data, effects, and projection matrix were combined into a stack object for the model fit.

The default priors in R-INLA were used for the hyperparameters in this analysis, namely: the variance and range. The model was fitted in R-INLA using the selected covariates and a spatial random field to predict the probabilities of a child testing positive at each observed location. Statistical significance to malaria parasite infection were explored by extracting the exponentiated covariates slopes.

### Model validation

The sampling method for the data resulted in some parts of the region not sampled and thus, without data leading to possible bias in prediction estimates. To confirm this, two approaches were used to evaluate the model: (1) data was randomly partitioned into 10-folds, with each fold having similar number of observations; (2) used spatial block cross-validation (CV) [33] to split the data randomly into 10-folds over the study area with each fold having different number of observations assigned as test and training data (Supplementary Table 5). These two approaches were to enable us to assess (1) how well the model fitted the data overall and (2) how well the model predicted the data in both sampled and unsampled areas. In the case of the random cross-validation, the model was trained 10 times. In each iteration, one-fold was held as a test set and the other nine were used as training set. In the spatial cross-validation, a set of data in particular blocks within each fold were randomly allocated as test and training sets for model prediction and model fitting respectively (Supplementary Fig. 3). In both approaches, we blinded the response variable in the test data by setting its value to ‘NA’ so that it did not use observed data for prediction. This step was repeated ten times, changing the fold ID in each iteration. The model performance was assessed by calculating the root mean squared error (RMSE) for each iteration in the model prediction and the model fitting (which we tagged as estimation). The computed RMSEs for the two approaches were then compared to make a judgement on the model performance. RMSE is an indication of the average deviation of the predictions from the actual observations and is one of the recommended metric for evaluating prevalence models [34]. A lower RSME is an indication of a model’s ability to predicted the observed data well [35].

### Model prediction

To explore spatial variation in malaria prevalence across the region and predict in unsampled areas, spatial points from one of the covariate rasters was extracted to get a spatial grid surface of 100 m resolution. This resulted in 434,280 points for prediction. Then 100 samples were extracted from the posterior of the full fitted model to predict malaria prevalence for children 6 months–10 years at 100 m for the study area. Mean predicted probabilities, standard deviations (SD), and credible intervals (CI) were calculated for each pixel and maps generated for the mean prediction and its associated SD, and CI.

### Exceedance probability

To assess the probability that the prevalence of malaria in some areas within the region exceeded 10%, we conducted an exceedance probability (EP) analysis using a 10% threshold. The 10% threshold was chosen to align with the WHO classification of < 10% parasite prevalence for low transmission settings [36, 37]. The EP account for the level of uncertainty around our prediction and provides useful information for making decisions. In this study, the EP was used to identify areas within low transmission areas that are likely to have malaria prevalence above 10% and, therefore, not appropriate to be targeted for malaria elimination interventions by the NMEP. To calculate the EP, the cumulative distribution function (CDF) of the predictions was assumed to be a beta distribution with values constrained between 0 and 1. Thus the EP was calculated as

$$P\left(Y>T\right)=1-P\left(Y\le T\right)=1-F(T)$$

where $P\left(Y>T\right)$ is the exceedance probability of predicted prevalence $Y$ exceeding a threshold $T$ and $F(T)$ is the CDF of the predicted values. The alpha and beta shape parameters were derived from the posterior means and standard deviation of the predictions. The alpha and beta parameters controls the shape of the distribution and sets them between 0 and 1, respectively [38].

## Results

### Descriptive analysis results

The final data of 12,371 children aged 6 months–10 years had more females (53%) than males (47%). The number of children who tested positive for RDT were 536 (4.3%) while 11,835 (95.6%) tested negative. More of the children (51%) were aged 6 month–5 years. RDT positivity was significantly higher in children 5–10 years (59%) compared with children less than 5 years (41%). Out of the 6333 children for which place of residence were recorded, majority (68%) lived in an urban area and the RDT results between the different areas of residence was statistically significant (p-value < 0.001). Proportionally, children residing in urban areas had higher RDT positive results (46%) compared with children in peri-urban (24%) and rural areas (30%). Table 1 highlights the demographic factors and RDT results in the dataset.

**Table 1:** Demographic parameters of study participants and their RDT results

			RDT results		
Variable	N	Overall, N = 12,371^a^	Negative, N = 11,835^a^	Positive, N = 536^a^	p-value^b^
Sex	12,371				0.3
Female		6548 (53%)	6276 (53%)	272 (51%)	
Male		5823 (47%)	5559 (47%)	264 (49%)	
Age group	12,371				< 0.001
0 < 5		6370 (51%)	6150 (52%)	220 (41%)	
5–10		6001 (49%)	5685 (48%)	316 (59%)	
Place of residence	6333				< 0.001
Rural		968 (15%)	889 (15%)	79 (30%)	
Peri_urban		1068 (17%)	1004 (17%)	64 (24%)	
Urban		4297 (68%)	4175 (69%)	122 (46%)	
Unknown		6038	5767	271	

^a^n (%)

^b^Pearson’s Chi-squared test

### Exploratory analysis results

#### Univariate and multivariate analysis

Supplementary Table 4 highlights the output of the univariate and multivariate analysis conducted to determine the association between the outcome variable (RDT positive) and the explanatory variables. In the univariate regression analysis, age group (5–10 years) and place of residence i.e., living in an urban area, were found to be significantly associated with malaria prevalence. Among the land cover covariates, water, tree and crop coverage were significantly associated with malaria prevalence. Land surface temperature bands 10 (LST_10) and 11 (LST_11), enhanced vegetation index (EVI), normalized difference vegetation index (NDVI), and tasselled cup greenness (TCG) were the climatic covariates significantly associated with the odds of malaria prevalence. All the urban covariates—settlement characteristics (built_c), building height (built_h), built surface (built_s), building volume (built_v), and population density (pop), were found to be significantly associated with the outcome. In the multivariate regression analysis, age group (5–10 years), place of residence (urban), LST_10, TCB, TCW and Pop significantly influence the odds of malaria prevalence in the region.

Mapping the distribution of RDT results over the study area (Supplementary Fig. 2), we observed that data was more clustered in the districts earmarked for elimination due to their population size compared with districts located outside the elimination zone.

### Principal component analysis (PCA) results

The result of the PCA conducted on the four urban covariates are shown in Supplementary Table 3. While PCA1 explained 82.5% of the variation in the covariates, PCA1-3 were selected, which cumulatively accounted for 99% of the variation.

### Geostatistical model results

The geostatistical model results showed that while predictors such as TCB and TCW were significantly associated with reduced odds of malaria prevalence, Trees and PC2 were associated with increased odds (Table 2). TCB was associated with a reduction in odds of malaria prevalence by 30% (OR 0.692, CI 0.527, 0.910) while TCW was associated with a reduction of 32% (OR 0.677, CI 0.484, 0.948). Conversely, Trees and PC2 were associated with an increase in odds of malaria prevalence by 16% (OR 1.163, CI 1.036, 1.1.306) and 16% (OR 1.156, CI 1.063, 1.258) respectively. Covariates such as water, built areas, bare ground, rangeland, LST_11, EVI, PC1, and PC3 were found not to significantly affect the odds of malaria prevalence in children in the Greater Accra region.

**Table 2:** Odds ratios of posterior mean of model parameters including standard deviation, lower and upper credible intervals

Parameter	Mean	Standard deviation	Lower CI	Upper CI
Intercept	0.079	1.415	0.040	0.155
Water	1.020	1.059	0.912	1.140
Trees	1.163	1.061	1.036	1.306
Built_area	1.087	1.243	0.709	1.666
Bareground	1.106	1.172	0.811	1.509
Rangeland	0.940	1.097	0.783	1.128
LST_11	0.809	1.120	0.648	1.010
EVI	1.193	1.140	0.923	1.541
TCB	0.692	1.150	0.527	0.910
TCW	0.677	1.187	0.484	0.948
PC1	0.972	1.042	0.896	1.054
PC2	1.156	1.044	1.063	1.258
PC3	0.878	1.073	0.764	1.009
Range	0.073	1.751	0.023	0.211
Variance	0.511	1.495	0.236	1.146

### Model validation

The average RSME for the final model was 0.199 (measured in the same units as the dependent variable—i.e., malaria infection prevalence expressed as a fraction between zero and 1) as shown in Supplementary Table 6. The RMSE of the prediction and estimation models were compared for both random and spatial blocking cross validations for each fold. Generally, the RMSE values from the two models are expected to be either similar or very close. The results showed that the prediction RMSEs are quite close to the estimation RMSE in the random cross validation. In some of the folds (2, 3, 4, and 6) there was lower RSME for the prediction model than the Estimation model. In the spatial blocking cross validation, a similar trend was observed however, three folds (1, 2, and 9), had much higher values in the prediction model than the estimation model. Overall, the RSMEs from the two approaches were closer to the average RSME of the final model.

### Prediction

**Fig. 2:**
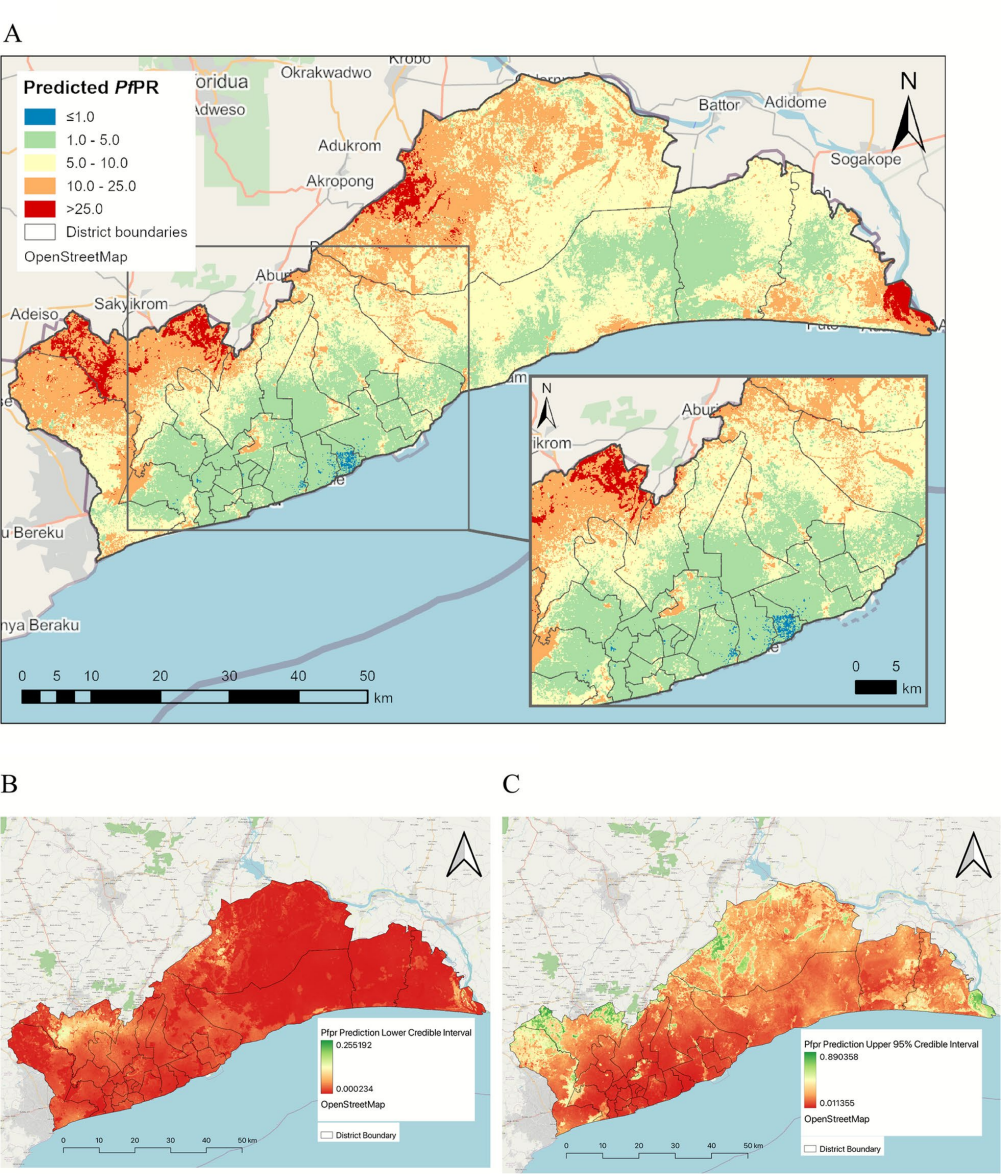
**A** Predicted mean malaria parasite prevalence in Children 6 month–10 years in Greater Accra region. Insert, predicted mean malaria prevalence in Districts earmarked for elimination. Parasite prevalence prediction lower credible interval (**B**) and Upper credible interval (**C**) among Children 6 months–10 years in Greater Accra Region

A prediction map of the malaria parasite prevalence in children under 10 years in Greater Accra region was produced at 100 m resolution (Supplementary Fig. 4) with the corresponding lower and upper 95% credible intervals (Fig. 2B, C). The predicted malaria prevalence ranged from 0.003 to 0.492. The prediction lower 95% credible interval ranged from 0.0 to 0.255 while the upper 95% credible interval ranged from 0.011 to 0.89. The estimated malaria prevalence was rescaled and re-categorized into operationally meaningful cut-offs (less than 1%, 1–5%, 5–10%, 10–25%, ≥ 25%) to align with thresholds used to stratify malaria risk in the National Malaria Elimination Strategic Plan. The prevalence map was then overlaid with district administrative boundaries to generate malaria prevalence map at district level (Fig. 2A). From the map, lower malaria prevalence was observed in the central part of the region compared with areas in the northern and eastern parts of the region. The northern part of the region which borders the Eastern region of Ghana and the south-eastern part of the region, had predicted parasite prevalence above 10% with some areas showing prevalence above 25%. Districts within the pre-elimination earmarked areas were found to have malaria parasite prevalence below 5% while districts outside that zone showed prevalence mostly above 5% as highlighted in the insert map. Whiles most of the pre-elimination areas showed prevalence below 5%, the northern part of districts like Ayawaso West, Ga East, La-Nkwantanang Madina and Adenta had prevalence above 10%.

Supplementary Fig. 5 is a map of the prediction standard deviation, which quantifies the degree of uncertainty around our estimate of the parasite prevalence for the region. The standard deviation map showed that the variation in our prediction was between 0 and 24.5%. Overall, the uncertainty of the prediction was low.

### Mapping exceedance probabilities

**Fig. 3:**
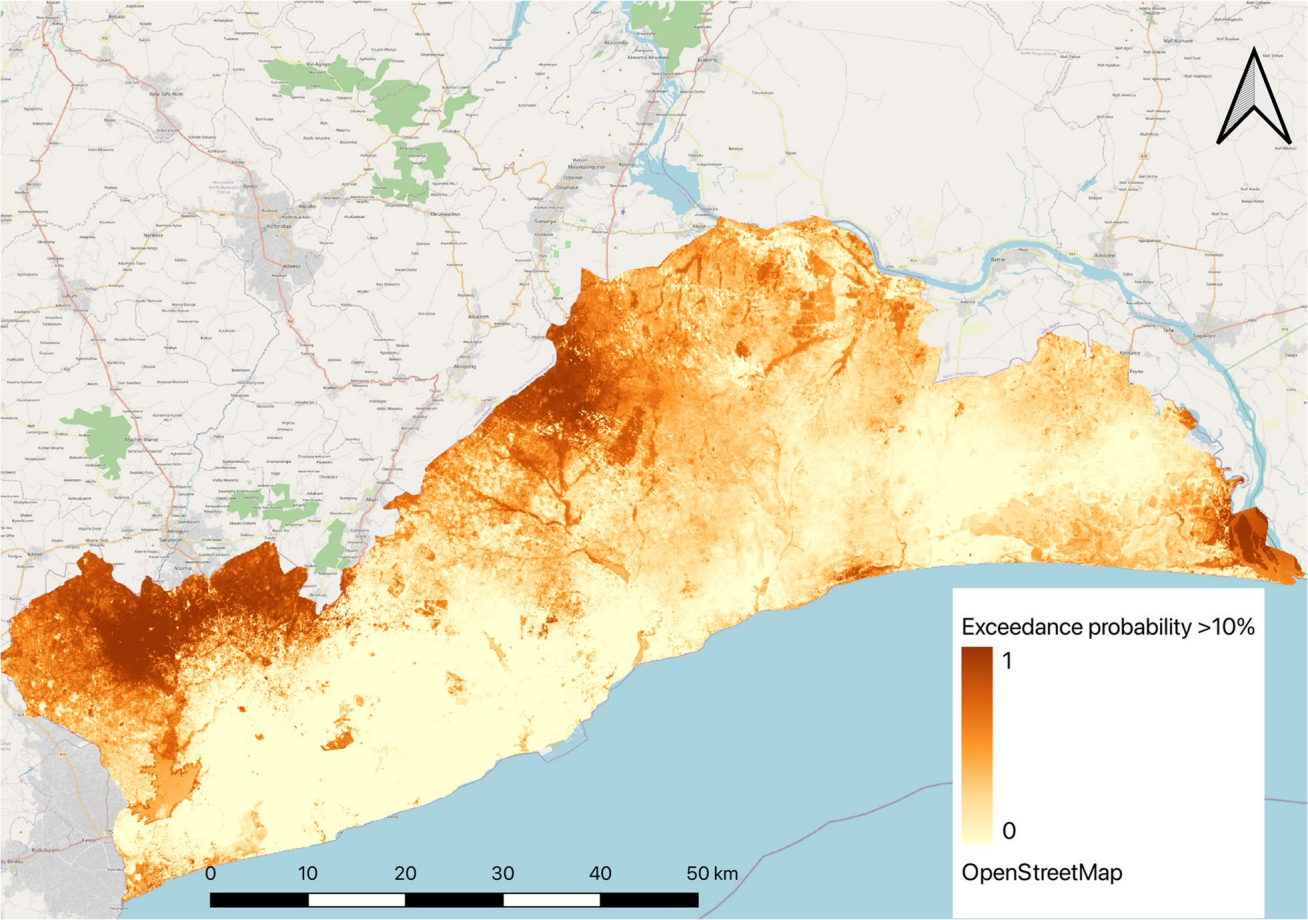
Exceedance probability of malaria parasite prevalence above 10% in children 6 months–10 years in Greater Accra region

The calculated exceedance probabilities of malaria prevalence being greater than 10% is shown in Fig. 3. The areas close to 0 on the scale, are more likely to have malaria prevalence below 10% and should be targeted for elimination activities. The areas close to 1 are thus, more likely to have prevalence above 10% and should be targeted for control interventions.

## Discussion

The study sought to achieve three specific objectives; (1) identify differences in malaria risk within and among districts; (2) explore factors associated with malaria prevalence in the region; and (3) provide information to better guide local planning of malaria interventions to deploy within the same district. The data collection coincided with a major raining season, allowing us to assess a period of peak malaria risk in the region. Moreover, every District in the region was sampled, making the predictions comparable and representative.

For the first objective, malaria prevalence for children 6 months–10 years and younger was predicted to be as low as 0.3% and as high as 49%. The West-central part of the region right through to the coastal and middle part of the region showed relatively low malaria prevalence compared with the upper/forest areas. The coastal and south-central part of the region are also more urbanized with high population density. This finding tends to agree with other studies which have reported lower malaria risk in urban areas than peri-urban and rural areas [3, 39]. As found by Kabaria et al. [40], the malaria risk within urban areas is heterogenous. The heterogeneity of malaria within the highly urban areas was evident in our prediction map where we observed malaria prevalence up to 25% in highly urbanized areas. One study have previously identified these high prevalence areas as slums [19]. This may be attributed to some species of mosquito like the Anopheles gambiae are which are increasingly adapting to polluted breeding sites in urban areas [2] or possibly An. stephensi.

It was observed that, predictions at 100 m spatial resolution, as used in this study, enabled us to capture finest spatial variations in neighbourhoods compared with similar studies that predicted at 1 km or 5 km [24, 39–41]. In rapidly growing urban settings in sub-Saharan Africa where there are profound differences in topography and socioeconomic factors, use of household or individual level data becomes imperative to capture difference in risk. Nuances at this lowest level become prohibitive when using cluster level data from DHS/MIS surveys where cluster locations are displaced by up to 5 km in rural clusters and up to 2 km in urban clusters [42]. This level of displacement can infer significant bias [43] especially in urban settings where malaria is highly focal and with marked variability in risk in short distances [3, 40].

While prevalence studies predict malaria risk in asymptomatic population, malaria incidence reported from health facilities gives an indication of malaria illness in the infected population. Two studies used the latter to predict malaria risk in the same study region [39, 44]. They observed seasonal pattern in malaria cases where it peaks in June/July, drops in September, and increases in October. The districts identified as having high malaria prevalence above 10% aligns with the findings from these studies. This work therefore compliments previous studies in the region and provides vital insights on malaria risk in the region using incidence and prevalence.

Model validation results show that we were able to predict malaria prevalence in Greater Accra with a high level of accuracy, evident from the low RMSE values we obtained in all the iterations in the two approaches. Additionally, the closeness of the prediction RMSE to the estimate RMSE, even though our test set did not include the observed values, indicates the model was able to predict well in unsampled areas. The wide age group from the data, 6 months–10 years, enabled us to profile malaria risk in one of the vulnerable groups. In most sub-Saharan countries lower prevalence have been observed in children under 5 years than older children as a result of chemoprevention strategies targeted at children aged less than 5 years [45].

Assessing factors associated with malaria prevalence in Greater Accra, four covariates were found to be significantly associated with the odds of a child testing positive for malaria. Tree coverage was the only environmental covariate found to increase the odds of malaria prevalence. In the prediction map, the high prevalence areas in the north-eastern and north-western side of the region are forest areas. This suggests that high forest cover increase the risk of malaria transmission, consistent with studies in Myanmar where population in settlements within 2km^2^ of natural forest had high malaria risk [46, 47]. This has been attributed to the land use practices of the population in those areas [47]. High tasselled cap brightness values –usually associated with partially covered soil or features such as concrete or asphalt, was found to reduce the odds of malaria prevalence. Covered, concrete or asphalted surfaces tend to provide very limited opportunity for breeding of mosquitoes. Though the individual urban covariates that influences malaria prevalence could not be determined, results of the non-spatial multivariate regression suggests that areas with high population density have reduced the odds of malaria prevalence. This finding is supported by Kabaria et al. [48], who found that malaria risk curve significantly reduces in areas with population densities > 1000 persons per km^2^. Factors explaining PC2 association with increased odds of malaria prevalence could not be explained in this study and will therefore need further research.

This study had a number of important limitations. The study did not account for temporal or seasonal variations in malaria risk in the region. Even though data collection for the original study coincided with the second peak season of malaria in the region, it would have been ideal if it captured the entire malaria transmission season to enable programme managers to consider optimal intervention timing.

The covariates used in this analysis were extracted for 2020, the year of the survey. Accra, like many urban areas in Africa is rapidly expanding with high population migration due to economic activities and therefore frequent changes in topography occur. Health system intervention like access to health services has improved since 2020 with expansion of health insurance as well as improvements and/or increase in health infrastructure. Increase in the number of health facilities and improvement in primary health services leading to reduced travel time and good access to care have been found to reduce malaria disease burden [49, 50]. There will be the need to repeat this kind of analysis using more recent survey data in scope and form and remote sensing data that reflect the current landscape in urban areas in the region. That notwithstanding, the results still represent a dramatic improvement in understanding the pattern and drivers of malaria risk in the region and reflect the most recent region-wide data available. The analysis will thus have numerous practical uses and can be leveraged to initiate discussions on spatial variations in malaria prevalence in the region.

Additionally, the analysis did not include household level factors such as type of building material used for the walls, floors, and ceiling as well as type of malaria intervention used in the household. It is envisaged that the addition of household factors will help improve understanding of the factors influencing the heterogeneity observed within highly urbanised areas as found in studies conducted elsewhere [3]. Lastly, the malaria prevalence estimates obtained are for children less than 10 years and may not represent prevalence for all age groups at risk of malaria in the region. The findings however, may reflect the ‘true’ malaria risk in the region as children, unlike adults, are more vulnerable due to lower immunity [23, 51].

The study results have provided useful information to support decision-making at both national and subnational levels on deployment of strategic malaria interventions. The observed range of prevalence Greater Accra region has implications for malaria interventions that should be deployed. While the latest DHS survey estimated a prevalence of 2% for the region [21], this study has identified districts and areas with prevalence as high as 49%. Children 5 years and older may have contributed more to high the prevalence in these areas than children less than 5 years as observed in our multivariate model (Supplementary Table 2). This assertion is in line with the findings of Dosoo et al. [22] and, therefore, interventions targeting older children should be deployed in the region. The exceedance probability map developed show areas within each district that are likely to have malaria prevalence above 10% and, therefore, needs control interventions. With the call by WHO for countries to stratify malaria risk and tailor interventions to where they are need most, this analysis provides a guide for the NMEP and district health managers to shift focus from one-size-fits-all approach and target appropriate interventions where they are needed most.

## Conclusion

The study has shown that, trees, tasselled cap brightness, tasselled cap wetness and urban covariates like population density, are associated with the odds of malaria prevalence among children 6 months–10 years in Greater Accra region. This work has identified the spatial heterogeneity of malaria in the region. The northern part of the region and south-eastern most part of the region has higher malaria risk compared with the central part. Within the districts earmarked for pre-elimination, hotspots have been identified where the probability of malaria prevalence above 10% is greater and therefore remains a threat to the elimination agenda. These areas need to be targeted for control interventions to drastically reduce the malaria risk. Additionally, this work will hopefully be a guide for the NMEP and health service managers in the district and the region to target the right intervention to areas based on the level of malaria risk. This will enhance better use of scarce resources to achieve maximum impact.

## Supplementary Information

Supplementary Material 1. Supplementary Fig. 1: Cascade of final number of observations used in the analysis. Supplementary Fig. 2: Distribution of RDT results of study participants. Supplementary Fig. 3: Spatial assignment of data into 10-folds for cross validation. Supplementary Fig. 4: Smooth predictive map of malaria prevalence for children 6 month to 10 years in Greater Accra Region. Supplementary Fig. 5: Standard deviation of predicted malaria prevalence in Children 6 month-10 years in Greater Accra region. Supplementary Table 1: Description of covariates, source, and spatial resolution. Supplementary Table 2: Final set of covariates and their VIF values. Supplementary Table 3: Number of observations for each fold for random and spatial blocking cross-validations. Supplementary Table 4: Univariate and Multivariate regression results. Supplementary Table 5: PCA results showing the relative PCA components. Supplementary Table 6: Root Mean Squared errorof prediction and estimation models for random and spatial blocking cross validations

## Data Availability

Available with restricted access: The sub-regional household prevalence study data that was used in this analysis are available from the Kintampo Health Research Centre, Ghana through the National Malaria Elimination Programme (NMEP), Ghana. Restrictions however, apply to the availability of these data, due to individual level identifiers such as geo-locations of sampled children which breaches ethics protocols and so are not publicly available. The data are, however, available upon request and with the permission of the NMEP Ghana.
