# Supplementary material for: Fine-scale spatial mapping of urban malaria prevalence for microstratification in an urban area of Ghana
Source: Malar J. 2025 Dec 18;25:50. doi: 10.1186/s12936-025-05724-9 (PMC12829291; doi:10.1186/s12936-025-05724-9)
Supplement: Supplementary file 1 — Supplementary Material 1. Supplementary Fig. 1: Cascade of final number of observations used in the analysis. Supplementary Fig. 2: Distribution of RDT results of study participants. Supplementary Fig. 3: Spatial assignment of data into 10-folds for cross validation. Supplementary Fig. 4: Smooth predictive map of malaria prevalence for children 6 month to 10 years in Greater Accra Region. Supplementary Fig. 5: Standard deviation of predicted malaria prevalence in Children 6 month-10 years in Greater Accra region. Supplementary Table 1: Description of covariates, source, and spatial resolution. Supplementary Table 2: Final set of covariates and their VIF values. Supplementary Table 3: Number of observations for each fold for random and spatial blocking cross-validations. Supplementary Table 4: Univariate and Multivariate regression results. Supplementary Table 5: PCA results showing the relative PCA components. Supplementary Table 6: Root Mean Squared errorof prediction and estimation models for random and spatial blocking cross validations [file 12936_2025_5724_MOESM1_ESM.docx]

Table of Contents

[Flow chart on final number of observation used in the analysis 2](#_Toc214634677)

[Covariates, sources and spatial resolutions 3](#_Toc214634678)

[Supplementary method section 3](#_Toc214634679)

[Covariate extraction 3](#_Toc214634680)

[Univariate and Multivariate regression results. 6](#_Toc214634681)

[Distribution of RDT results across the region 8](#_Toc214634682)

[PCA results and PCA components. 8](#_Toc214634683)

[Covariate selection via Variable Inflation factor (VIF) 9](#_Toc214634684)

[Random and spatial blocking cross-validations 9](#_Toc214634685)

[Spatial blocking of data 9](#_Toc214634686)

[Results 10](#_Toc214634687)

[Model validation 10](#_Toc214634688)

[Prediction map of malaria prevalence in Greater Accra region 11](#_Toc214634689)

Tables

[Supplementary table 1: Description of covariates, source, and spatial resolution 3](#_Toc214634646)

[Supplementary table 2: Table showing the number of observations for each variable, the odds ratio (OR), lower and upper credible intervals (95% CI) and the p-value for both univariate and multivariable regression. 6](#_Toc214634647)

[Supplementary table 3: PCA results showing the relative PCA components. 8](#_Toc214634648)

[Supplementary table 4: Final set of covariates and their VIF values 9](#_Toc214634649)

[Table 5: Number of observations for each fold for random and spatial blocking cross-validations 9](#_Toc214634650)

[Supplementary table 6: Root Mean Squared error (RMSE) of prediction and estimation models for random and spatial blocking cross validations. 10](#_Toc214634651)

Table of figures

[Supplementary figure 1: Flow chart of final number of observations use for the analysis 2](#_Toc214634658)

[Supplementary figure 2: Distribution of RDT results of study participants. Blue dots indicate negative mRDT results while red dots indicate positive result 8](#_Toc214634659)

[Supplementary figure 3: Spatial assignment of data into 10-folds for cross validation. Red dots denote samples assigned as test set and blue dots denote samples assigned as train set. 10](#_Toc214634660)

[Supplementary figure 4: Smooth predictive map of malaria prevalence for children 6 month to 10 years in Greater Accra Region 11](#_Toc214634661)

[Supplementary figure 5: Standard deviation (C) of predicted malaria prevalence in Children 6month-10years in Greater Accra region 12](#_Toc214634662)

# Flow chart on final number of observation used in the analysis

Supplementary figure 1: Flow chart of final number of observations use for the analysis

17,035 children 6months to 10 years enrolled.

29 records without RDT results

17,006 records with RDT results

15,628 records with geocoordinates

1,378 records without geocoordinates

12,371 records with household level geocoordinates

3,257 records with geocoordinates for the centroid of the community

Number of children in unique households

10654 HHs – 1 child

729 HHs – 2 children

79 HHs – 3 children

4 HHs – 4 children

1 HH – 6 children

Supplementary figure 1: Cascade of final number of observations used in the analysis.

# Covariates, sources and spatial resolutions

Supplementary table 1: Description of covariates, source, and spatial resolution

| Covariate | Description | Sources | Spatial resolution |
| --- | --- | --- | --- |
| **Land cover classes** | | | |
| Water | Water | LANDSAT/LC08/C02/T1 | 30m |
| Trees | Trees | LANDSAT/LC08/C02/T1 | 30m |
| Crops | Crops | LANDSAT/LC08/C02/T1 | 30m |
| Built-area | Built area | LANDSAT/LC08/C02/T1 | 30m |
| Flooded_veg | Flooded vegetation | LANDSAT/LC08/C02/T1 | 30m |
| Bareground | Bare ground | LANDSAT/LC08/C02/T1 | 30m |
| Rangeland | Rangeland | LANDSAT/LC08/C02/T1 | 30m |
| **Environmental/ climatic** | | | |
| EVI | Enhanced vegetation index | MODIS derivative(1) | 30m |
| NDVI | Normalized difference vegetation index | Landsat 8-USGS | 30m |
| LSTemp_10 | Land surface temperature band 10 | MODIS derivative | 30m |
| LSTemp_11 | Land surface temperature band 11 | MODIS derivative | 30m |
| TCB | Tasselled cap brightness; measure of reflectance | MODIS derivative | 30m |
| TCG | Tasselled cap greenness | MODIS derivative | 30m |
| TCW | Tasselled cap wetness | MODIS derivative | 30m |
| **Urban covariates** | | | |
| GHS-BUILT-H | GHS built-up height | Global Human settlement layer (GHSL) | 100m |
| GHS-BUILT-S | GHS built-up surface | Global Human settlement layer (GHSL) | 100m |
| GHS-BUILT-V | GHS built-up volume | Global Human settlement layer (GHSL) | 100m |
| GHS-BUILT-C | GHS built-up characteristics | Global Human settlement layer (GHSL) | 100m |
| GHS-POP | GHS population grid (R2023) | Global Human settlement layer (GHSL) | 100m |

Supplementary figure 1: Cascade of final number of observations used in the analysis.

# Supplementary method section

## Covariate extraction

We derived global images for all variables, except the urban variables, from Landsat 8 data at 30m spatial resolution and aggregated them to 100m resolution using Google Earth Engine(2). To create cloud-free images, we employed the Earth Engine Simple Composite algorithm, which selects the 10 least cloudy pixels for each location, generating a median value. This process was performed for each of the four seasons, and a yearly average was then computed to minimize the influence of seasonally biased cloud cover.

Seven land cover classes were selected based on their relevance to the study area's topography. These classes include water, trees, crops, built area, rangeland, bare ground, and flooded vegetation. For each class, we calculated the proportion of the pixel covered by that specific land cover over the study area boundaries.

For environmental and climatic variables, we included Tasselled Cap Brightness (TCB), Tasselled Cap Wetness (TCW), Tasselled Cap Greenness (TCG), Enhanced Vegetation Index (EVI), Normalised Difference Vegetation Index (NDVI) and Land surface temperature (LST). NDVI captures the availability of transient and partly dense vegetation. EVI acts differently and is more sensitive to areas with high-biomass specific to some anopheles species(3). TCW measures the soil and vegetation moisture and maps permanent wetlands which act as persistent sources for mosquito breeding enabling us to identify stable malaria hotspots. This is different from the ‘water’ - land cover variable which measures the proportion of areas covered by waterbodies – as water surface enables laying of eggs by the mosquito. TCB, a measure of overall reflectance of an area, measures changes in land-use like deforestation or urbanization which influence mosquito habitats. Areas with high brightness and low vegetation cover can indicate drier or disturbed environments that may still harbor mosquito breeding sites, particularly in areas with poor water management. Several studies have demonstrated that the thermal characteristic of the soil, vegetation and atmosphere influence the breeding of the *Anopheles* mosquito(4). LST measure the exchange of water and heat on the earth’s surface with the atmosphere across different bands(5). While band 10 is sensitive to soil and vegetation, band 11 is more influenced by high humidity.

EVI and NDVI were calculated using standard formulas:

- $EVI=2.5*\left( \frac{Band5-Band4}{Band5+6\times Band4-7.5 \times Band2+1} \right)$(1)
- $NDVI =\frac{\left( Band 5 - Band 4 \right)}{\left( Band 5 + Band 4 \right)}$ (6)

TCB, TCW, TCG were derived using coefficients from Baig, M. H. A., et al. (2014) (7). LST, which influences the rate and timing of plant growth (8) was calculated using the simplified version of the Planck's law-based LST calculation that is commonly used in remote sensing and thermal imagery analysis (9).

$LST=\frac{B_{T}}{(1+w\left( \frac{B_{T}}{p} \right)*\ln\left( e \right))}$

where$B_{T}$ is Brightness Temperature (measured from thermal infrared remote sensing data)$w$ is wavelength of the emitted radiance,$p$ is Planck's constant, and $e$ is Emissivity (surface emissivity). We calculated LST separately for bands 10 (LSTemp_10) and bands 11 (LSTemp_11) from Landsat and included emissivity from MODIS11A2 v061 using band 31 and band 2 for LSTemp_10 and LSTemp_11 calculations respectively (10).

We obtained the urban variables from the Global Human Settlement Layer (GHSL) website(11). The GHSL data included GHS population grid(12), GHS built-up volume grid(13), GHS built-up surface grid(14), GHS settlement Characteristics(15), and GHS building height grid(16).

## Univariate and Multivariate regression results.

Supplementary table 2: Table showing the number of observations for each variable, the odds ratio (OR), lower and upper credible intervals (95% CI) and the p-value for both univariate and multivariable regression.

|  | **Univariate** | | | | **Multivariable** | | |
| --- | --- | --- | --- | --- | --- | --- | --- |
| **Characteristic** | **N** | **OR** | **95% CI** | **p-value** | **OR** | **95% CI** | **p-value** |
| Sex | 12,371 |  |  |  |  |  |  |
| Female |  | — | — |  | — | — |  |
| Male |  | 1.10 | 0.92, 1.30 | 0.3 | 1.07 | 0.83, 1.37 | 0.6 |
| Place of residence | 6,333 |  |  |  |  |  |  |
| Rural |  | — | — |  | — | — |  |
| Peri_urban |  | 0.72 | 0.51, 1.01 | 0.057 | 0.75 | 0.49, 1.15 | 0.2 |
| Urban |  | 0.33 | 0.25, 0.44 | <0.001 | 0.59 | 0.40, 0.88 | 0.009 |
| Age group | 12,371 |  |  |  |  |  |  |
| 0<5 |  | — | — |  | — | — |  |
| 5-10 |  | 1.55 | 1.30, 1.85 | <0.001 | 1.47 | 1.14, 1.90 | 0.003 |
| Water | 12,354 | 1.19 | 1.07, 1.30 | <0.001 | 1.01 | 0.89, 1.14 | 0.9 |
| Trees | 12,354 | 1.50 | 1.37, 1.62 | <0.001 | 1.17 | 1.02, 1.33 | 0.022 |
| Crops | 12,354 | 1.28 | 1.07, 1.50 | 0.003 | 0.70 | 0.29, 1.16 | 0.3 |
| Flood_veg | 12,354 | 1.09 | 0.66, 1.50 | 0.7 | 0.93 | 0.55, 1.35 | 0.7 |
| Built_area | 12,354 | 0.93 | 0.76, 1.19 | 0.5 | 1.08 | 0.66, 2.15 | 0.8 |
| Bareground | 12,354 | 0.97 | 0.69, 1.22 | 0.8 | 1.33 | 0.83, 1.85 | 0.14 |
| Rangeland | 12,354 | 0.98 | 0.87, 1.10 | 0.8 | 0.96 | 0.72, 1.25 | 0.8 |
| LST_10 | 12,354 | 0.57 | 0.51, 0.64 | <0.001 | 0.38 | 0.12, 1.16 | 0.088 |
| LST_11 | 12,354 | 0.61 | 0.55, 0.69 | <0.001 | 1.76 | 0.59, 5.30 | 0.3 |
| EVI | 12,354 | 2.10 | 1.86, 2.37 | <0.001 | 2.03 | 0.35, 11.7 | 0.4 |
| NDVI | 12,354 | 2.00 | 1.78, 2.25 | <0.001 | 0.80 | 0.11, 5.90 | 0.8 |
| TCB | 12,354 | 1.12 | 0.98, 1.28 | 0.086 | 0.48 | 0.28, 0.78 | 0.005 |
| TCG | 12,354 | 1.90 | 1.70, 2.11 | <0.001 | 0.85 | 0.26, 2.79 | 0.8 |
| TCW | 12,354 | 1.01 | 0.86, 1.18 | >0.9 | 0.54 | 0.30, 0.92 | 0.028 |
| Built_c | 12,354 | 0.92 | 0.87, 0.98 | 0.009 | 1.05 | 0.95, 1.18 | 0.3 |
| Built_h | 12,354 | 0.65 | 0.60, 0.71 | <0.001 | 4,336 | 1.27, 19,969,839,063 | 0.2 |
| Built_s | 12,354 | 0.62 | 0.57, 0.68 | <0.001 | 0.86 | 0.59, 1.25 | 0.4 |
| Built_v | 12,354 | 0.65 | 0.60, 0.70 | <0.001 | 0.00 | 0.00, 0.96 | 0.2 |
| Pop | 12,354 | 0.76 | 0.72, 0.80 | <0.001 | 0.85 | 0.76, 0.95 | 0.004 |
| Abbreviations: CI = Confidence Interval, OR = Odds Ratio | | | | | | | |

## Distribution of RDT results across the region


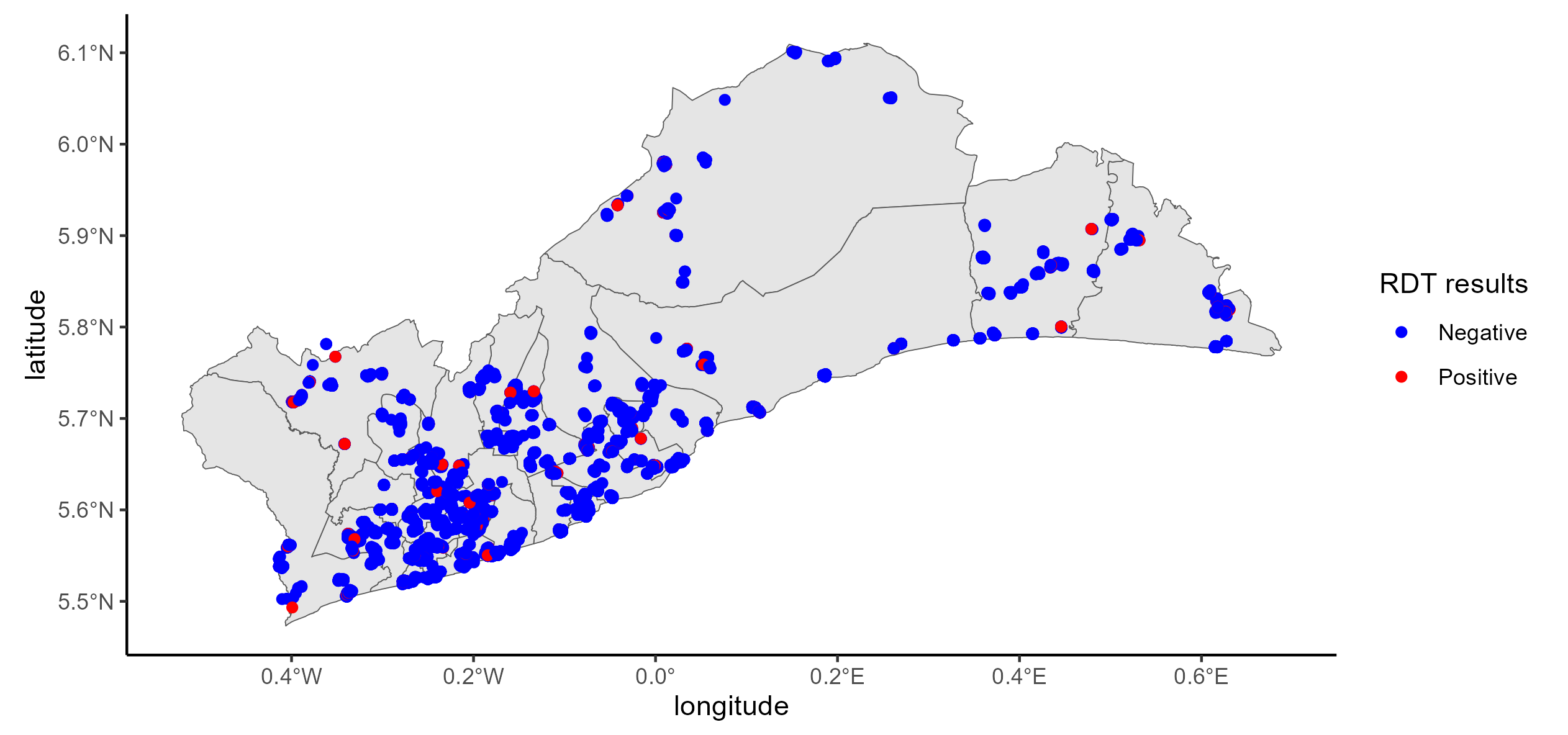


Supplementary figure 2: Distribution of RDT results of study participants. Blue dots indicate negative mRDT results while red dots indicate positive result

## PCA results and PCA components.

Table shows the standard deviation, the proportion of variation and cumulative proportion for each principal component.

Supplementary table 3: PCA results showing the relative PCA components.

| PCA components | PCA1 | PCA2 | PCA3 | PCA4 |
| --- | --- | --- | --- | --- |
| Standard deviation | 1.7773 | 0.6380 | 0.4844 | 0.1714 |
| Proportion of variation | 0.8248 | 0.1063 | 0.0613 | 0.0076 |
| Cumulative Proportion | 0.8248 | 0.9311 | 0.9923 | 1.000 |

## Covariate selection via Variable Inflation factor (VIF)

Supplementary table 4: Final set of covariates and their VIF values

| Covariates | VIF value |
| --- | --- |
| Water | 1.060 |
| Trees | 1.131 |
| Built_area | 2.301 |
| Bareground | 1.117 |
| Rangeland | 2.223 |
| LST_11 | 1.564 |
| EVI | 22.976 |
| TCB | 4.343 |
| TCW | 4.131 |
| PC1 | 3.03 |
| PC2 | 1.184 |
| PC3 | 1.721 |

## Random and spatial blocking cross-validations

Table 5: Number of observations for each fold for random and spatial blocking cross-validations

| Fold ID | Random cross validation | Spatial blocking cross validation | |
| --- | --- | --- | --- |
|  |  | Test set | Train set |
| 1 | 1238 | 2456 | 9915 |
| 2 | 1237 | 376 | 11995 |
| 3 | 1237 | 2399 | 9972 |
| 4 | 1237 | 203 | 12168 |
| 5 | 1237 | 349 | 12022 |
| 6 | 1237 | 533 | 11838 |
| 7 | 1237 | 1005 | 11366 |
| 8 | 1237 | 4024 | 8347 |
| 9 | 1237 | 438 | 11933 |
| 10 | 1237 | 588 | 11783 |

## Spatial blocking of data


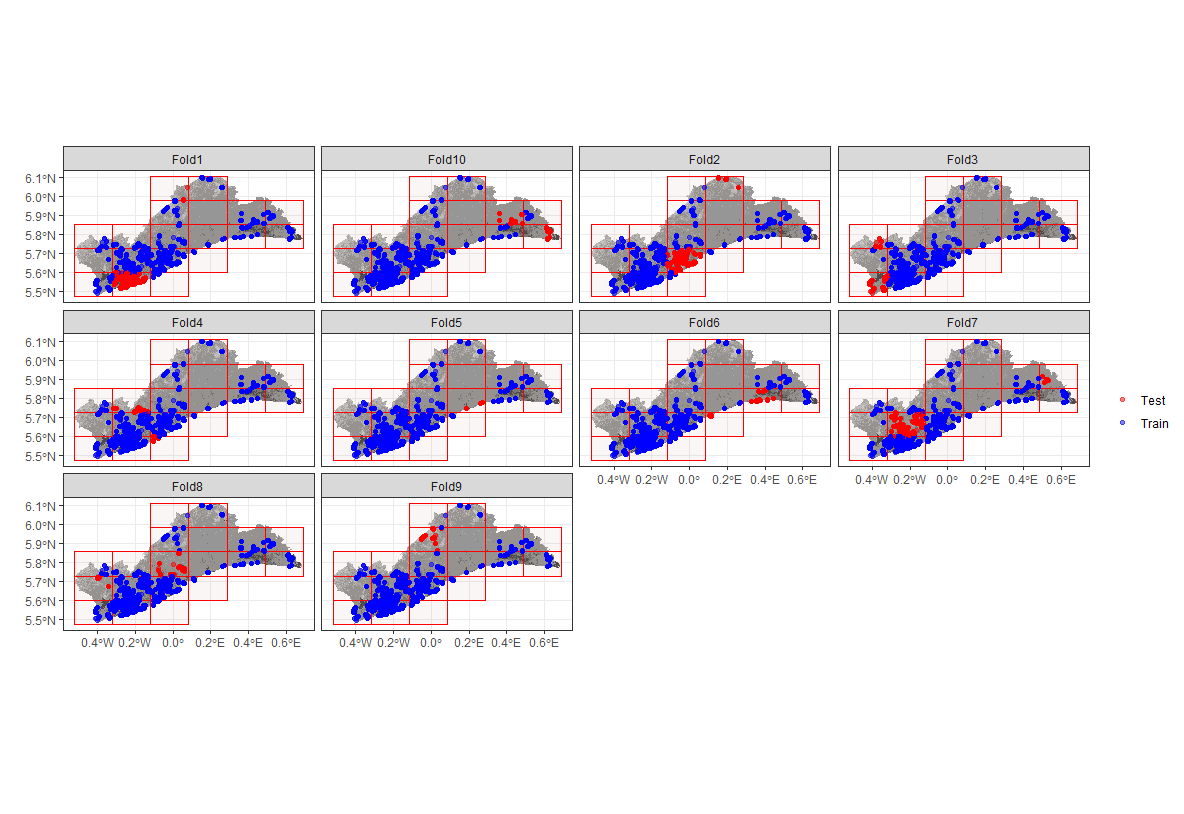


Supplementary figure 3: Spatial assignment of data into 10-folds for cross validation. Red dots denote samples assigned as test set and blue dots denote samples assigned as train set.

# *Results*

## Model validation

Supplementary table 6: Root Mean Squared error (RMSE) of prediction and estimation models for random and spatial blocking cross validations.

| Random cross validation | Spatial blocking cross validation |
| --- | --- |
| \| Fold \| Prediction RMSE \| Estimation MSE \| \| --- \| --- \| --- \| \| 1 \| 0.2106 \| 0.1979 \| \| 2 \| 0.1815 \| 0.2010 \| \| 3 \| 0.1936 \| 0.1998 \| \| 4 \| 0.1906 \| 0.2001 \| \| 5 \| 0.2004 \| 0.1989 \| \| 6 \| 0.1925 \| 0.1999 \| \| 7 \| 0.2174 \| 0.1971 \| \| 8 \| 0.2034 \| 0.1986 \| \| 9 \| 0.2066 \| 0.1984 \| \| 10 \| 0.2036 \| 0.1986 \| | \| Spatial block \| Prediction RMSE \| Estimation RMSE \| \| --- \| --- \| --- \| \| 1 \| 0.3004 \| 0.1945 \| \| 2 \| 0.3648 \| 0.1907 \| \| 3 \| 0.1816 \| 0.2030 \| \| 4 \| 0.2065 \| 0.1979 \| \| 5 \| 0.2072 \| 0.1987 \| \| 6 \| 0.2199 \| 0.1980 \| \| 7 \| 0.1554 \| 0.2015 \| \| 8 \| 0.1911 \| 0.1993 \| \| 9 \| 0.2664 \| 0.1965 \| \| 10 \| 0.1635 \| 0.2149 \| |

## Prediction map of malaria prevalence in Greater Accra region


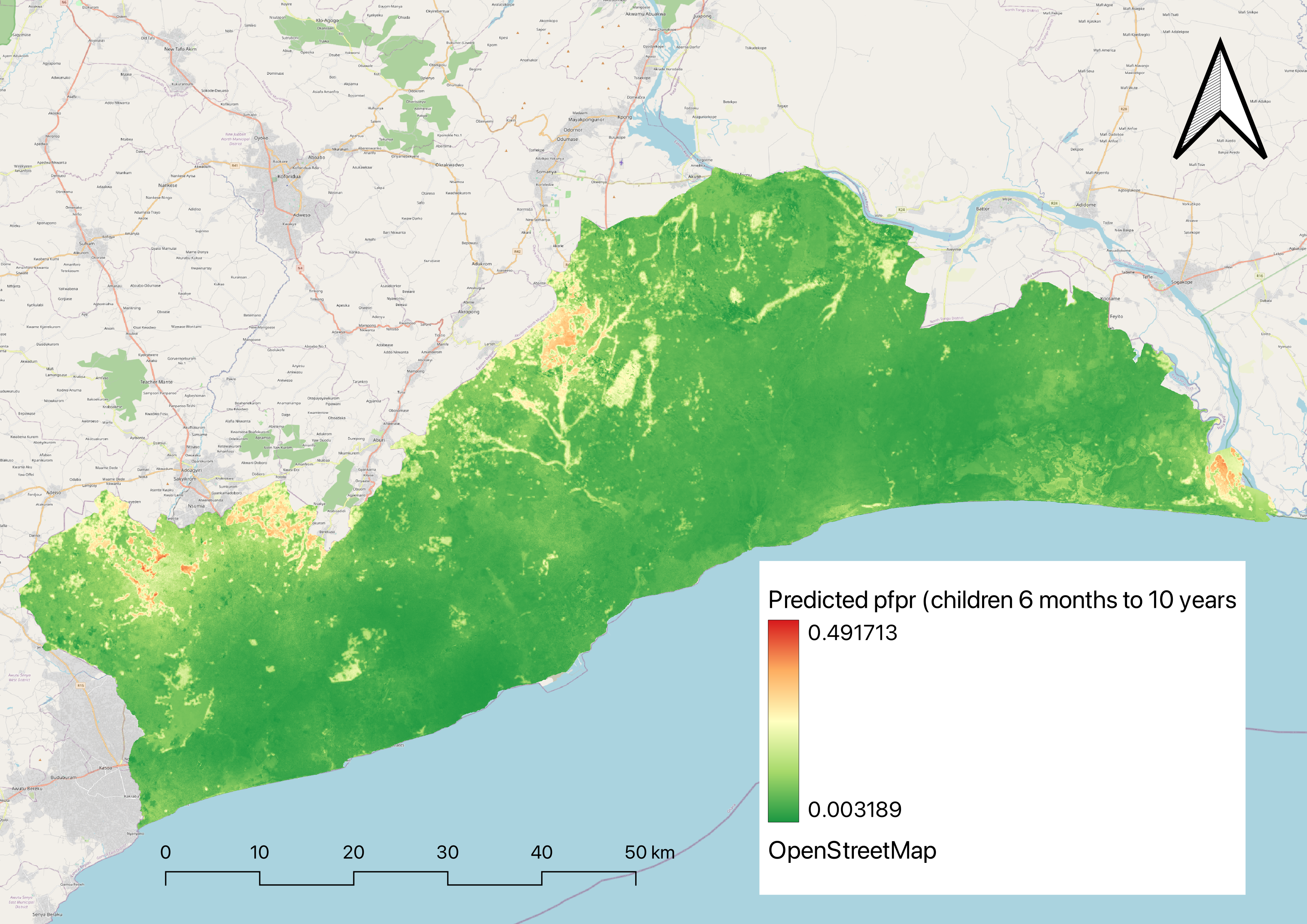


Supplementary figure 4: Smooth predictive map of malaria prevalence for children 6 month to 10 years in Greater Accra Region

Prediction standard deviation


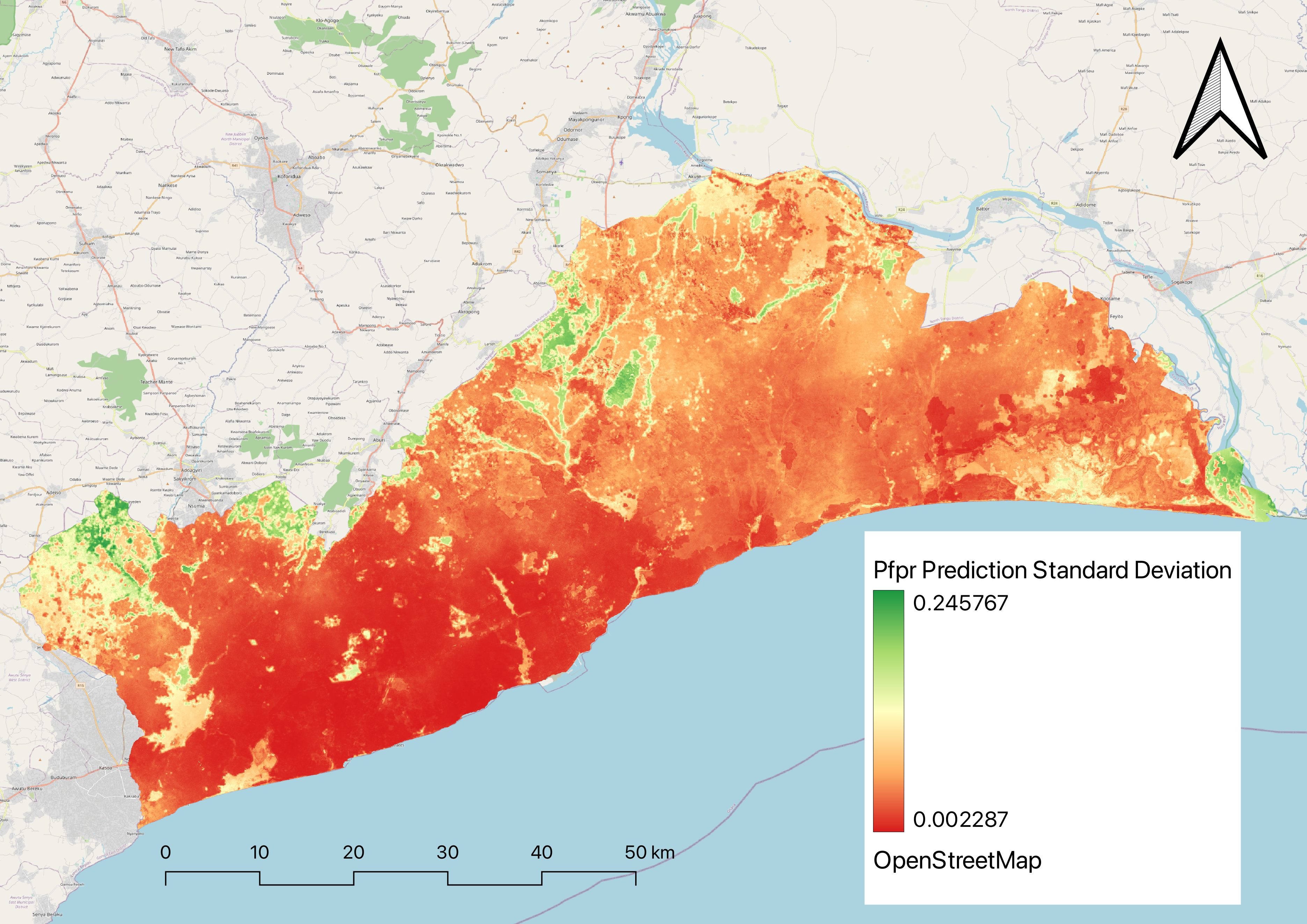


Supplementary figure 5: Standard deviation (C) of predicted malaria prevalence in Children 6month-10years in Greater Accra region

References

1. Landsat Enhanced Vegetation Index | U.S. Geological Survey [Internet]. [cited 2024 Sept 19]. Available from: https://www.usgs.gov/landsat-missions/landsat-enhanced-vegetation-index

2. Google for Developers [Internet]. [cited 2024 Oct 29]. USGS Landsat 8 Collection 2 Tier 1 Raw Scenes | Earth Engine Data Catalog. Available from: https://developers.google.com/earth-engine/datasets/catalog/LANDSAT_LC08_C02_T1

3. L J. Enhanced Vegetation Index (EVI): A Modern Approach to Vegetation Health Monitoring [Internet]. Geospatial Artificial Intelligence | GeoAI. 2024 [cited 2025 Nov 17]. Available from: https://geoai.au/enhanced-vegetation-index-evi-a-modern-approach-to-vegetation-health-monitoring/

4. Youssefi F, Zoej MJV, Hanafi-Bojd AA, Dariane AB, Khaki M, Safdarinezhad A, et al. Temporal Monitoring and Predicting of the Abundance of Malaria Vectors Using Time Series Analysis of Remote Sensing Data through Google Earth Engine. Sensors (Basel). 2022 Mar 2;22(5):1942.

5. Kikon N, Kumar D, Ahmed SA. Analysing transition of land surface temperature and derived indices with respect to elevation values in Kohima Saddar. GeoJournal. 2022 Oct 1;87(4):821–46.

6. Landsat Normalized Difference Vegetation Index | U.S. Geological Survey [Internet]. [cited 2024 Sept 24]. Available from: https://www.usgs.gov/landsat-missions/landsat-normalized-difference-vegetation-index

7. Baig MHA, Zhang L, Shuai T, Tong Q. Derivation of a tasselled cap transformation based on Landsat 8 at-satellite reflectance. Remote Sensing Letters. 2014 May 4;5(5):423–31.

8. ESA Climate Office [Internet]. [cited 2024 Sept 24]. Land Surface Temperature. Available from: https://climate.esa.int/en/projects/land-surface-temperature/

9. Product Tutorial on Land Surface Temperature (LST) [Internet]. [cited 2024 Sept 24]. Available from: https://resources.eumetrain.org/data/4/460/navmenu.php?tab=2&page=2.0.0

10. Wan Z, Hook S, Hulley G. MODIS/Terra Land Surface Temperature/Emissivity 8-Day L3 Global 1km SIN Grid V061 [Internet]. NASA EOSDIS Land Processes Distributed Active Archive Center; 2021 [cited 2024 Sept 24]. Available from: https://lpdaac.usgs.gov/products/mod11a2v061/

11. Global Human Settlement - Visualisations of the GHSL datasets - European Commission [Internet]. [cited 2024 Sept 11]. Available from: https://human-settlement.emergency.copernicus.eu/visLanding.php

12. Schiavina M. Global Human Settlement - GHS-POP_GLOBE_R2023A - European Commission [Internet]. [cited 2024 Sept 16]. Available from: https://human-settlement.emergency.copernicus.eu/ghs_pop2023.php

13. Pesaresi M, Politis P. GHS-BUILT-V R2023A - GHS built-up volume grids derived from joint assessment of Sentinel2, Landsat, and global DEM data, multitemporal (1975-2030) [Internet]. European Commission, Joint Research Centre (JRC); 2023 [cited 2024 Sept 16]. Available from: http://data.europa.eu/89h/ab2f107a-03cd-47a3-85e5-139d8ec63283

14. Pesaresi M. GHS-BUILT-S R2023A - GHS built-up surface grid, derived from Sentinel2 composite and Landsat, multitemporal (1975-2030) [Internet]. European Commission, Joint Research Centre (JRC); 2023 [cited 2024 Sept 16]. Available from: http://data.europa.eu/89h/9f06f36f-4b11-47ec-abb0-4f8b7b1d72ea

15. Pesaresi M, Politis P. GHS-BUILT-C R2023A - GHS Settlement Characteristics, derived from Sentinel2 composite (2018) and other GHS R2023A data [Internet]. European Commission, Joint Research Centre (JRC); 2023 [cited 2024 Sept 16]. Available from: http://data.europa.eu/89h/3c60ddf6-0586-4190-854b-f6aa0edc2a30

16. Pesaresi M. GHS-BUILT-H R2023A - GHS building height, derived from AW3D30, SRTM30, and Sentinel2 composite (2018) [Internet]. European Commission, Joint Research Centre (JRC); 2023 [cited 2024 Sept 16]. Available from: http://data.europa.eu/89h/85005901-3a49-48dd-9d19-6261354f56fe
